# Effect of the Ketone Body Beta-Hydroxybutyrate on the Innate Defense Capability of Primary Bovine Mammary Epithelial Cells

**DOI:** 10.1371/journal.pone.0157774

**Published:** 2016-06-16

**Authors:** Maria Hillreiner, Claudia Flinspach, Michael W. Pfaffl, Heike Kliem

**Affiliations:** Chair of Animal Physiology and Immunology, Technische Universität München, Freising, Germany; Humboldt-University Berlin, GERMANY

## Abstract

Negative energy balance and ketosis are thought to cause impaired immune function and to increase the risk of clinical mastitis in dairy cows. The present *in vitro* study aimed to investigate the effect of elevated levels of the predominant ketone body β-hydroxybutyrate on the innate defense capability of primary bovine mammary epithelial cells (pbMEC) challenged with the mastitis pathogen *Escherichia coli* (*E*. *coli*). Therefore, pbMEC of healthy dairy cows in mid- lactation were isolated from milk and challenged in culture with 3 mM BHBA and *E*. *coli*. pbMEC stimulated with *E*. *coli* for 6 h or 30 h showed an up-regulation of several innate immune genes, whereas co-stimulation of pbMEC with 3 mM BHBA and *E*. *coli* resulted in the down-regulation of CCL2, SAA3, LF and C3 gene expression compared to the challenge with solely the bacterial stimulus. These results indicated that increased BHBA concentrations may be partially responsible for the higher mastitis susceptibility of dairy cows in early lactation. Elevated levels of BHBA in blood and milk during negative energy balance and ketosis are likely to impair innate immune function in the bovine mammary gland by attenuating the expression of a broad range of innate immune genes.

## Introduction

High-producing dairy cows often experience a state of negative energy balance (NEB) during the first weeks of lactation. Milk production rapidly increases after calving, so that the cows require more energy for maintenance, growth and milk production than they are able to obtain through feed intake and digestion [1–3]. To compensate for the deficiency in energy supply in early lactation, body fat reserves are mobilized resulting in an increase of the plasma concentration of non-esterified fatty acids (NEFA) [4,5]. NEFA are taken up by the liver and further processed via the β-oxidation pathway in mitochondria, where acetyl-CoA is formed, which enters the tricarboxylic acid cycle (TCA cycle) to form citrate by condensation with oxaloacetate. In order to generate essential glucose and carbohydrates in NEB, gluconeogenesis occurs in the liver using oxaloacetate as main substrate. This leads to an insufficient availability of oxaloacetate for the removal of acetyl-CoA and hence an accumulation of acetyl-CoA. Alternatively, acetyl-CoA is metabolized via ketogenesis to the ketone bodies acetoacetate, β-hydroxybutyrate (BHBA) and acetone [1]. Therefore, cows in NEB show increased concentrations of ketone bodies in their blood and milk. The predominant circulating ketone body in dairy cattle that contributes to the development of subclinical or even clinical ketosis is BHBA [2]. Subclinical ketosis is characterized by elevated plasma concentrations of ketone bodies in the absence of the clinical signs of ketosis [6] and is defined at a plasma concentration of 1.4 mM BHBA [5,7], whereby the threshold for clinical ketosis is defined at a plasma concentration of 2 to 3 mM BHBA [5]. Cows suffering from clinical ketosis show signs of indigestion resulting in decreased feed intake, body weight and milk production. In addition, they can be lethargic or abnormally agitated [4]. It has already been discussed that NEB and ketosis might be linked to an increased risk of clinical mastitis in dairy cows, especially in the early phase of lactation. The major mastitis pathogens that cause mastitis are either gram-positive germs, like *Staphylococcus aureus* or gram-negative germs like *Escherichia coli* (*E*. *coli*). In case of an *E*. *coli* induced mastitis, the milk producing parenchyma, the milk collecting cistern and the teat show acute symptoms of inflammation, elevated body temperature, decreased milk production and elevated somatic cell counts (SCC) [6]. Mastitis affects dairy cow health and welfare, but is also one of the most costly diseases in the dairy industry due to reduced milk yield and quality [8]. Since primary bovine mammary epithelial cells (pbMEC) are known to be part of the innate immune system of the bovine mammary gland [9], we used a 3D cell culture approach to investigate the effect of elevated BHBA levels on the innate defense capability of pbMEC challenged with the mastitis pathogen *E*. *coli*. pbMEC represent the first cellular line of defense, after a pathogen manages to cross the teat barrier, therefore pbMEC express transepithelial receptors, like the toll-like receptors (TLR) on their cell surface [10]. Through the activation of the TLR-pathway by pathogen associated molecular patterns, pbMEC are able to induce a downstream signaling cascade, resulting in the activation of the transcription factor NFĸB and hence in the production of chemotactic molecules like pro-inflammatory chemokines, cytokines and acute phase proteins [11,12]. Thus, the recruitment of other immune cells, like leucocytes and macrophages to the site of infection is one of the main tasks of pbMEC in innate immunity. Therefore, pbMEC of healthy dairy cows in mid-lactation were isolated from fresh milk and challenged with 3 mM BHBA and *E*. *coli*. The approach aimed to stimulate the metabolic state of ketosis and, in addition, an inflammation of the mammary epithelium *in vitro*. Results should elucidate the influence of ketosis on the innate immune response of the bovine mammary gland to mastitis.

## Materials and Methods

### Cultivation of pbMEC

The pbMEC were isolated from the milk of six healthy, first lactating Brown Swiss cows (Veitshof Research Station, Technische Universität München, Freising, Germany) in mid-lactation (100–200 days in lactation). Cows with a somatic cell count (SCC) below 200,000 cells per milliliter milk, were considered healthy. The cell isolation was basically conducted as described in previous studies [12,13], with slight changes in the washing procedure. In brief, milk cells were harvested (10 min, 1850 × g), the cell pellet was washed with 1x HBSS buffer (Sigma-Aldrich, Saint Louis, USA) supplemented with antibiotics and antimycotics (penicillin/streptomycin, amphotericin B, Sigma-Aldrich) and the cell suspension was filtered twice through filters with different pore sizes (100 μm, 40 μm, Greiner Bio-One GmbH, Frickenhausen, Germany). The cells were then re-suspended in 4 ml of pre-warmed DMEM-F12 Ham medium (Sigma-Aldrich) supplemented with ITS liquid media supplement, antibiotics, antimycotics (Sigma-Aldrich) and FBS (gibco^®^ Lifetechnologies GmbH, Darmstadt, Germany) (proliferation medium), and seeded in one well of a coated (2.4 mg/ml Matrigel^®^, Corning Inc., Corning, NY) 6-well culture plate. The cells were cultivated at 37°C, 5% CO_2_ and 90% humidity. The pbMEC were sub-cultivated twice with 0.25% trypsin-EDTA solution (Sigma-Aldrich), before they were cryopreserved in liquid nitrogen.

### Immunocytochemistry

The pbMEC of all animals were seeded at a density of 1.5×10^4^ cells per well in 8-well LabTec chamber slides (LAB-Tec, Nunc, GmbH, Langenselbold, Germany) and provided with proliferation medium. At a confluency of 70–80% the medium and the culture chambers were removed and the cells fixed in ice cold methanol/acetone (1:1) for 10 min. Immunocytochemistry (IC) was conducted with the monoclonal mouse anti-cytokeratin pan antibody clone C-11 (1:400 in PBST, Sigma-Aldrich), as described earlier [13] [14].

### Mycoplasma Test

The PCR Mycoplasma Test Kit (AppliChem GmbH, Darmstadt, Germany) was used. Cell culture supernatants were sampled for each animal and stored at– 80°C until further processing. The analysis was conducted according to the manufacturer’s instructions.

### Imitation of the ketotic state and immune stimulation with *E*. *coli*

**Fig 1:**
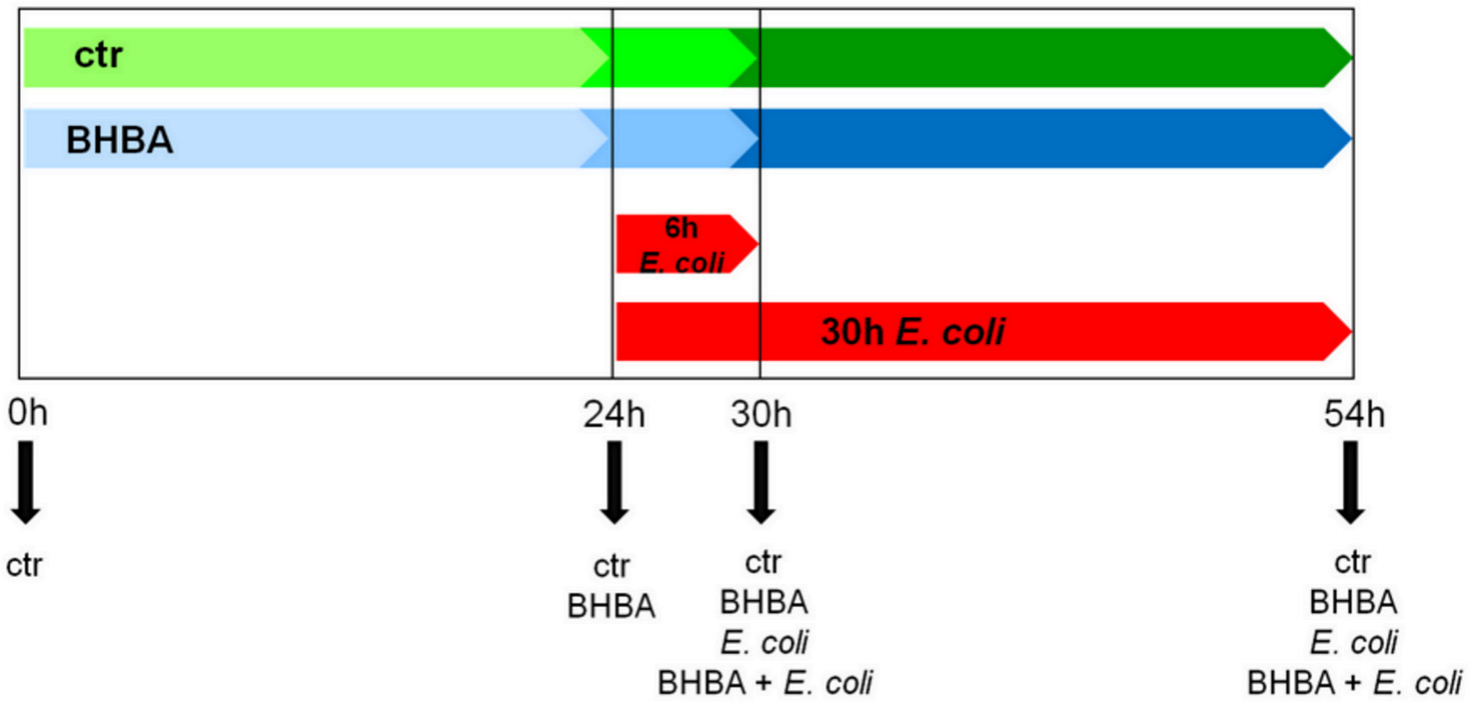
Treatment scheme of the pbMEC *in vitro*. The green arrow indicates the untreated control samples that were taken at every relevant time-point (0 h, 24 h, 30 h and 54 h). The blue arrow indicates the BHBA treatment time-points. The pbMEC that were only treated with 3 mM BHBA were also sampled at every relevant time-point (24 h, 30 h and 54 h). The red arrows indicate the two treatment intervals for the *E*. *coli* treatment (6 h and 30 h). It can be clearly seen that within the co-stimulated pbMEC, a 24 h adaptation phase to 3 mM BHBA preceded the bacterial treatment, so that pbMEC stimulated with *E*. *coli* for 6 h, obtained a total BHBA treatment period for 30 h and pbMEC stimulated with E. coli for 30 h obtained a total BHBA treatment period of 54 h.

Treatment with BHBA and *E*. *coli* was performed in duplicate. The pbMEC were seeded at a density of 2×10^4^ cells per well in 6-well plates coated with 2.4 mg/ml Matrigel^®^ (Corning Inc., Corning, NY). For the *E*. *coli* treatment, three counting wells were seeded per animal. The pbMEC on those wells were trypsinized with a solution of 0.25% trypsin-EDTA (Sigma-Aldrich) upon reaching confluency and counted (TC10™ Automated Cell Counter, Bio-Rad Laboratories GmbH) using the life-dead staining with 0.4% trypan blue (Bio-Rad Laboratories GmbH, Munich, Germany). The mean value of the cell count served as benchmark for the cell number in all other experimental wells and was the basis for calculating the multiplicity of infection (MOI). After reaching 70–80% confluency, the proliferation medium was replaced with DMEM/F12 Ham medium supplemented with only ITS (Sigma-Aldrich) (challenge medium). After 24 h, the medium was removed and the first samples were taken (0 h time-point). The other cells were treated using different approaches. Control cells were left untreated and were further incubated in a challenge medium for 24 h, 30 h and 54 h. Cells which should be treated with 3 mM BHBA (Sigma-Aldrich), were treated with challenge medium supplemented with 3 mM BHBA for additional 24 h, 30 h and 54 h. For cells to be treated either only with *E*. *coli* or with both *E*. *coli* and 3 mM BHBA, pbMEC were infected with a multiplicity of infection (MOI) of 30 colony forming units per cultured cell. The infected pbMEC were further cultivated for 6 h and 30 h with *E*. *coli* alone, or with *E*. *coli* and BHBA (30 h BHBA & 6 h *E*. *coli* and 54 h BHBA & 30 h *E*. *coli*) (Fig 1). The cell culture samples were lysed in Qiazol (Qiagen, Hilden, Germany) and stored at -80°C until further analysis. For each treatment time-point, cell culture supernatants were also sampled and stored at -80°C.

### RNA extraction and reverse transcription (RT)

Total RNA, including mRNA and miRNA, was extracted with the miRNeasy Micro Kit (Qiagen) according to the manufacturer’s instructions with slight modifications. An additional incubation for 5 min with buffer RPE was added to the standard protocol in order to reduce contaminations of the RNA with guanidine thiocyanate. The NanoDrop 1000 photometer (Peqlab, Erlangen, Germany) was used to verify RNA concentration and purity, whereas the 2100 Bioanalyzer and the RNA 6000 Nano Kit (Agilent Technologies, Waldbronn, Germany) were used to determine RNA integrity. The analysis was conducted following the manufacturer’s instructions. Isolated RNA was stored at -80°C until further processing. The master mix for the reverse transcription of 400 ng RNA to cDNA contained the following components in a total volume of 20 μl: 0.5 mM Oligo-d(T) primers (Fermentas, St Leon-Rot, Germany), 0.5 M dNTPs, 5x buffer, 100U M-MLV H(—) reverse transcriptase (Promega, Mannheim, Germany) and 2.5 μM random hexamer primers (Invitrogen Life Technologies, Darmstadt, Germany). The cDNA was diluted 1:1 to a final concentration of 10 ng/μl. RNA isolated from bovine spleen and bovine udder tissue was used as positive control. Furthermore, a negative control and a non-template control (NTC) were carried along with the RT-PCR. The T-Personal Thermocycler (Biometra, Göttingen, Germany) was used to conduct the RT-PCR according to the following protocol: Annealing: 21°C, 10 min, transcription phase: 48°C, 50 min, degrading phase: 90°C, 2 min. cDNA was stored at– 20°C until further analysis.

### RT-qPCR primer design

24 specific primer pairs for *Bos taurus* were designed using Primer3web version 4.0.0 [15,16], based on published bovine nucleic acid sequences of the National Center for Biotechnology Information gene database (NCBI, National Library of Medicine, Bethesda MD), and ordered from Sigma-Aldrich. Amongst primers targeting the genes involved in the innate immune response, 4 primers targeted the reference genes ACTG1, GAPDH, YWHAZ and KRT8 (Table 1).

**Table 1:** Primer for RT-qPCR measurements.

Gene name	NCBI reference sequence number	Primer sequence (5’ -> 3’)	L^1^ [bp]
		Forward	
		Reverse	
***TLR pathway***			
Toll-like receptor 4 (*TLR4*)	NM_174198.6	TGCTGGCTGCAAAAAGTATG	213
		TTACGGCTTTTGTGGAAACC	
Myeloid differentiation primary response gene (*MYD88*)	NM_001014382.2	CTGCAAAGCAAGGAATGTGA	122
		AGGATGCTGGGGAACTCTTT	
V-rel reticuloendotheliosis viral oncogene homolog A (avian) (NF-kappa-B p65 subunit) (*RELA*)	NM_001080242.2	ACAGCTTTCAGAACCTGGGG	140
		GACGGCATTCAGGTCGTAG	
***Complement system***			
Complement component 3 (*C3*)	NM_001040469	AAGTTCATCACCCACATCAAG	191
		CACTGTTTCTGGTTCTCCTC	
***Chemokines***			
Chemokine (C-C motif) ligand 2 (*CCL2*)	NM_174006.2	TCTCGCTGCAACATGAAGGT	121
		TATAGCAGCAGGCGACTTGG	
Chemokine (C-C motif) ligand 20 (*CCL20*)	NM_174263.2	CTTGTGGGCTTCACACAGC	115
		GTTTCACCCACTTCTTCTTTGG	
Interleukin 8 (*CXCL8*)	NM_173925.2	AAGAATGAGTACAGAACTTCGATGC	160
		GTTTAGGCAGACCTCGTTTCC	
***Inflammatory cytokines***			
Interleukin 6 (*IL6*)	NM_173923.2	TGGTGATGACTTCTGCTTTCC	109
		AGAGCTTCGGTTTTCTCTGG	
Tumor necrosis factor α (*TNFα*)	NM_173966.2	CCACGTTGTAGCCGACATC	108
		ACCACCAGCTGGTTGTCTTC	
***Acute phase proteins / danger associated molecular pattern molecules***			
Serum amyloid A3 (*SAA3*)	NM_001242573.1	CACGGGCATCATTTTCTGCTT	179
		GGGCAGCGTCATAGTTTCCA	
Haptoglobin (*HP*)	NM_001040470.1	AATGAACGATGGCTCCTCAC	176
		TTGATGAGCCCAATGTCTACC	
S100 calcium binding protein A9 (*S100A9*)	NM_001046328.1	CTGGTGCAAAAAGAGCTGC	128
		AGCATAATGAACTCCTCGAAGC	
***Antimicrobial peptides***			
Lactoferrin (*LF*)	NM_180998.2	CGAAGTGTGGATGGCAAGGAA	215
		TTCAAGGTGGTCAAGTAGCGG	
Lysozyme 1 K (*LYZ1*)	NM_001077829.1	AAGAAACTTGGATTGGATGGC	185
		ACTGCTTTTGGGGTTTTGC	
Tracheal antimicrobial peptide (*TAP*)	NM_174776.1	AGGAGTAGGAAATCCTGTAAGCTGTGT	113
		AGCATTTTACTGCCCGCCCGA	
Lingual antimicrobial peptide (*LAP*)	NM_203435.3	AGAAATTCTCAAAGCTGCCG	107
		CAGCATTTTACTTGGGCTCC	
***Lactogenesis***			
Signal transducer and activator of transcription 5 (*STAT5*)	NM_001012673.1	GTGAAGCCACAGATCAAGCA	176
		TCGAATTCTCCATCCTGGTC	
β-casein (CSN2)^2^	NM_181008.2	GGCTATGGCTCCTAAGCACA	163
		AGTTGGAGGAAGAGGCTGGT	
ĸ-casein (CSN3)^3^	NM_174294.1	GGAGCCTAAAACCCACAGACA	151
		CAGCACAACTTTGGAAGGGC	
***Others***			
Myxovirus (influenza virus) resistance 2 (mouse) (*MX2*)	NM_173941.2	CTTCAGAGACGCCTCAGTCG	232
		TGAAGCAGCCAGGAATAGTG	
***Reference genes***			
Actin, gamma 1 (*ACTG1*)	NM_001033618	AACTCCATCATGAAGTGTGAC	234
		GATCCACATCTGCTGGAAGG	
Glyceraldehyd-3-phosphate dehydrogenase (*GAPDH*)	NM_001034034.1	GTCTTCACTACCATGGAGAAGG	197
		TCATGGATGACCTTGGCCAG	
Tyrosine 3-monoxygenase/tryptophan 5-monoxygenase activation protein, zeta polypeptide (*YWHAZ*)	NM_174814.2	CAGGCTGAGCGATATGATGA	141
		GACCCTCCAAGATGACCTAC	
Cytokeratin 8 (*KRT8*)	NM_001033610	TGGTGGAGGACTTCAAGACC	215
		CGTGTCAGAAATCTGAGACTGC	

^1^ L: Length in base pair

^2^ Tm: 62°C

^3^ Tm: 54°C

### Expression profiling via RT-qPCR

The RT-qPCR reactions were conducted on the CFX384™ Real-Time System (Bio-Rad Laboratories GmbH). 10 ng/μl cDNA were mixed together with a master mix consisting of SsoFast™ EvaGreen^®^ Supermix (Bio-Rad Laboratories GmbH), VisiBlue™ qPCR mix colorant (TATAA Biocenter, Gothenburg, Sweden), RNase/DNase-free DEPC water and 20 μM of each Primer. The RT-qPCR was conducted on the CFX384™ Real-Time System with the following protocol: Activation of the DNA polymerase: 95°C, 30 sec, and 40 cycles of cDNA denaturation: 95°C, 5 sec and primer annealing and elongation: 54°C, 60°C, 62°C (*), 5 sec (* primer specific annealing temperature, Table 1). Primer specificity was verified by checking the melting curves of the RT-qPCR products.

### Data pre-processing and data analysis

The cycle of quantification (Cq) was automatically detected by the CFX Manager Software version 2.1 (Bio-Rad Laboratories GmbH). Using the geNorm tool in GenEx 5.4.4 (MultiD; Gothenburg, Sweden), raw Cq values were processed to identify stably expressed reference genes for target gene normalization. Fold changes of gene expression were determined according to the 2^^(-ddCq)^ method according to [17] for each sample. Statistics were calculated using SigmaPlot 13.0 (Systat Software GmbH). The dCq values were checked for a normal distribution, before the paired t-test was conducted. In the case of abnormally distributed data, a signed rank test was used. Regulation of gene expression was regarded as differentially expressed for p ≤ 0.05 in a paired t-test on dCq values between the treatment group and the corresponding control group or between two different treatments. When distinct changes in gene expression are mentioned, a p-value between 0.05 < p < 0.10 was calculated. The minimum information for the publication of quantitative real-time PCR experiments (MIQE) Guidelines [18] was considered during the entire RT-qPCR quantification workflow.

### ELISA measurements

The amount of secreted protein into the cell culture supernatant was evaluated for lactoferrin (LF) and CCL2. The protein content of LF in the cell culture supernatants was determined using the lactoferrin ELISA established at our institute and described in earlier studies [13]. Slight variations were applied to adapt the protocol to the low concentration of LF obtained in cell culture supernatants. Therefore, the standard curve for LF (colostrum isolate, Sigma-Aldrich) ranged from 12.5 ng/100 μl– 0.049 ng/100 μl and was diluted in phosphate buffered saline-Tween buffer (PBS-T, 1 g/l Tween 20, Merck Chemicals GmbH). For the ELISA measurements, 100 μl of the diluted standard and the undiluted cell culture supernatants, were applied to multiwell plates (96-well, Maxisorp, Nunc^®^, Sigma-Aldrich, Saint Louis, USA). Additionally 50 μl of the LF antibody (Ak8836, BE Ak8836, BE 08.092009) (1:300000 in PBST) were added to each well of the microplate. The ELISA plate was incubated at 4°C and left slightly shaking overnight. Afterwards, the ELISA plate was set to room temperature for 30 min before 6 ng/100 μl biotinylated LF was applied and incubated on a shaker for 45 min at room temperature. The microplate was then washed four times with PBST and 100 μl streptavidin-HRP working solution (Roche Applied Science, Basel, Switzerland) diluted 1:20000 in PBST was added and incubated for 15 min protected from light. After four additional washing steps, the HRP substrate reaction was started [19] and stopped after 40 min in the dark with 2 M H_2_SO_4_ before the substrate reaction was evaluated at 450 nm using a Microplate Reader (Sunrise^™^, Tecan Group Ltd., Männedorf, Switzerland). The mean value of the LF concentration was determined using the OD values of the standard curve within the linear range.

For the determination of the CCL2 amount in cell culture supernatants, the bovine CCL2 VetSet^™^ ELISA Development Kit (Kingfisher Biotech, St Paul, USA) was used. The ELISA was conducted according to the manufacturer’s instructions, with slight changes. As the challenge medium did not contain any carrier protein, the samples and the standard diluent (challenge medium) were supplemented with a solution of 4% BSA (Sigma-Aldrich) in DPBS, so that finally 1% BSA was present in the cell culture samples and the standard diluent. The substrate reaction was induced by the addition of TMB substrate and stopped after 30 min with 0.18 M H_2_SO_4_ (ELISA Accessory Pack, Kingfisher Biotech). The mean value of the CCL2 concentration was determined using the OD values of the standard curve within the linear range. For the statistical analysis of the treatment effect on CCL2 and LF protein secretion, the mean values of the *E*.*coli* treated group were compared to the mean values of the co-stimulated treatment group, using a paired t-test.

## Results

### Confirmation of the epithelial character and purity of the pbMEC cultures

**Fig 2:**
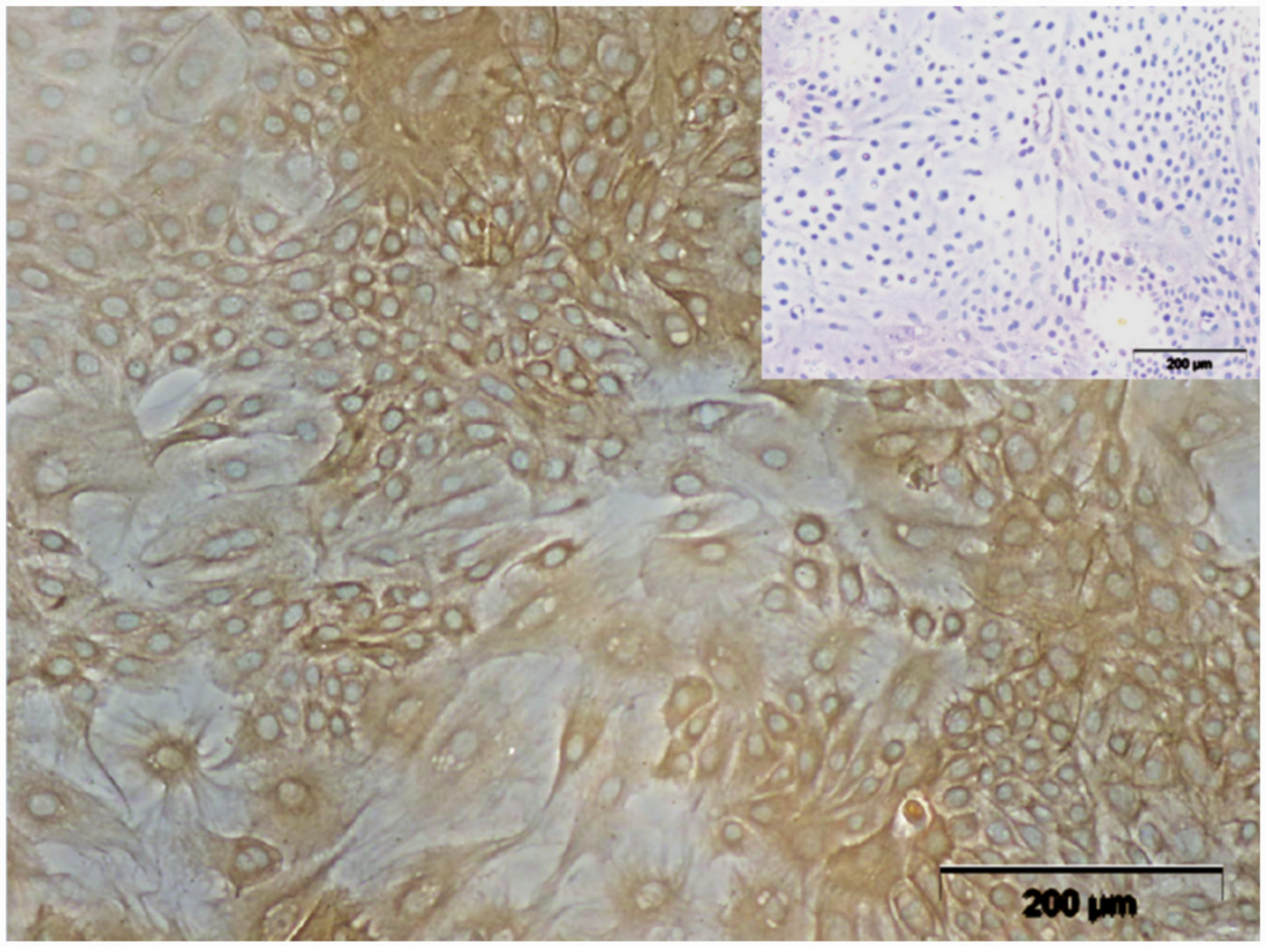
IC of pbMEC. The typical cobblestone-like morphology and the epithelial character were confirmed, by the positive cytokeratin staining, the insert shows the negative control. [Leica light microscope, magnification x200].

It was proven that none of the pbMEC cultures used in this experiment were contaminated with mycoplasma. Furthermore, an IC confirmed the epithelial character of the cells, as all pbMEC cultures were positively stained with the monoclonal mouse anti-cytokeratin pan antibody clone C-11 and showed the typical cobblestone-like morphology (Fig 2).

### Effects of the immune stimulation on pbMEC in ketotic and non-ketotic state

The ketone body BHBA itself had only slight effects on the gene expression of CCL20, CXCL8, IL8, TNFα, LYZ1, TAP, C3, RELA and LF (Table 2). However, due to a normal t-test, a distinct up-regulation in the gene expression of the genes coding for CCL20, LYZ1 and TNFα, and a significant induction of gene expression for the genes coding for CXCL8, IL6, RELA and TAP, could be detected (Table 2). Only the gene coding for LF showed a significant down-regulation 30 h after treatment start (Table 2). All other genes evaluated in this study were not altered by the treatment with 3 mM BHBA itself (Table 2). Treatment with the gram-negative pathogen *E*. *coli*, however, had a great influence on the gene expression of nearly all immune genes investigated in this study. In both cases, when cells were only treated with *E*. *coli* or co-stimulated with *E*. *coli* and 3 mM BHBA, a strong induction of the immune gene expression could be detected. Regarding the gene expression of genes coding for components of the TLR pathway, no differential gene expression could be detected for MyD88 when compared to the untreated control (Table 2). Only the TLR4 gene expression showed a significant up-regulation 30 h after treatment start (Table 2). The gene expression of RELA, the NF-kappa-B p65 subunit, was significantly up-regulated when the pbMEC were only treated with *E*. *coli* for 6 h. The genes coding for the chemokines, however, were strongly induced by the *E*. *coli* treatment and co-stimulation with *E*. *coli* and 3 mM BHBA (Table 2). Therefore, CCL2 and CCL20 gene expression was significantly up-regulated during the 6 h and the 30 h treatment time-point. By contrast, the gene expression of CXCL8 was also strongly induced, however, a significant increase in the gene expression could only be detected 30 h post-infection. The gene expression of the two pro-inflammatory cytokines IL6 and TNFα was also highly significantly induced 30 h post infection in the *E*. *coli* treated and in the co-stimulated samples (Table 2). Regarding the antimicrobial peptides LF, LYZ1 and TAP, a distinct as well as significant up-regulation of the gene expression could be detected through the *E*. *coli* treatment and co-stimulation (Table 2). Stimulation with the mastitis pathogen *E*. *coli* also induced a significant up-regulation of the gene expression of the gene coding for the acute phase protein SAA3 after 6 h and in the co-stimulatory experiment 30 h after treatment start (Table 2). The danger associated molecular pattern molecule S100A9 was distinctly and highly significant altered by the *E*. *coli* treatment and co-stimulation with *E*. *coli* and 3 mM BHBA. No effect of the treatment with *E*. *coli* or the co-stimulation with *E*. *coli* and 3 mM BHBA could be detected on the gene expression of the gene responsible for the induction of lactogenesis, STAT5, and for the gene coding for the milk protein κ-casein (CSN3) (Table 2).

**Table 2:** Fold changes in gene expression upon immune stimulation.

	Time-point											
	30h						54h					
	Treatment						Treatment					
Genes	BHBA^1^		*E*.*coli*^2^		BHBA + *E*.*coli*^3^		BHBA^4^		*E*.*coli*^5^		BHBA + *E*.*coli*^6^	
***Chemokines***												
CCL2												
Fold	0.89		7.56	***	6.29	**	1.16		94.72	***	58.12	***
SEM	0.10		2.00		2.12		0.26		46.64		27.19	
CCL20												
Fold	1.39		49.49	**	46.16	**	1.86	+	205.78	***	185.22	***
SEM	0.34		21.32		23.99		0.30		89.43		77.31	
CXCL8												
Fold	2.12	*	10.13		7.66		1.92	*	29.29	***	30.67	***
SEM	0.44		2.16		1.54		0.49		8.68		9.43	
***Inflammatory cytokines***												
IL6												
Fold	1.38	*	2.71		2.86		1.52		5.72	***	6.07	***
SEM	0.14		0.49		0.61		0.28		1.99		1.86	
TNFα												
Fold	1.24		17.62		17.04		1.85	+	49.68	***	44.36	***
SEM	0.22		6.06		7.84		0.34		17.76		15.32	
***Antimicrobial peptides***												
LF												
Fold	1.14		5.49	**	2.39	+	0.71	*	9.59	***	4.99	**
SEM	0.23		1.85		0.68		0.12		4.32		2.04	
LYZ1												
Fold	1.13		2.79	+	2.21	*	2.22	+	25.92	**	16.59	**
SEM	0.18		0.76		0.59		0.55		9.19		6.75	
TAP												
Fold	1.22		3.70	+	3.39	*	2.59	*	66.83	**	38.11	**
SEM	0.44		1.12		1.26		0.59		34.84		15.95	
***Acute phase proteins***												
SAA3												
Fold	0.94		35.19	***	31.38	**	1.83		2302.14		738.94	***
SEM	0.19		11.15		13.04		0.35		1183.19		361.50	
***Complement system***												
C3												
Fold	1.02		3.12	**	2.04		0.77	+	10.54	**	3.43	***
SEM	0.16		0.60		0.62		0.16		4.72		0.84	
***TLR signaling***												
TLR4												
Fold	1.05		1.36	*	1.04		0.90		1.17		1.20	
SEM	0.09		0.14		0.11		0.10		0.11		0.11	
MYD88												
Fold	1.07		1.08		1.02		0.98		1.01		0.99	
SEM	0.08		0.09		0.10		0.09		0.10		0.10	
RELA												
Fold	1.37	**	1.32	*	1.14		1.17		1.42		1.33	
SEM	0.11		0.15		0.12		0.23		0.23		0.32	
***Others***												
MX2												
Fold	1.81		3.89	**	5.08	*	2.15		13.42	***	8.20	***
SEM	0.39		0.79		1.66		0.88		4.16		2.50	
S100A9												
Fold	1.28		3.88	+	2.94	*	1.43		11.94	**	9.73	**
SEM	0.46		1.52		1.00		0.24		4.33		4.07	
***Lactogenesis***												
STAT5												
Fold	1.64		1.55		1.84		1.40		1.07		1.25	
SEM	0.48		0.63		0.73		0.26		0.14		0.14	
CSN3												
Fold	1.05		1.40		1.64		1.31		2.36		1.46	
SEM	0.18		0.35		0.57		0.24		0.69		0.37	

The treatment effect was statistically evaluated using a paired t-test.

^1^ 30h BHBA treatment

^2^ 6h *E*. *coli* treatment

^3^ 30h BHBA and 6h *E*. *coli* treatment

^4^ 54h BHBA treatment

^5^ 30h *E*. *coli* treatment

^6^ 54h BHBA and 30h *E*. *coli* treatment

^+^ Trend 0.1 ≤ p ≤ 0.05

* p ≤ 0.05

** p ≤ 0.01

*** p ≤ 0.001

### Differences in the gene expression of pbMEC through the co-stimulation of pbMEC with *E*.*coli* and 3 mM BHBA or only *E*. *coli*

**Fig 3:**
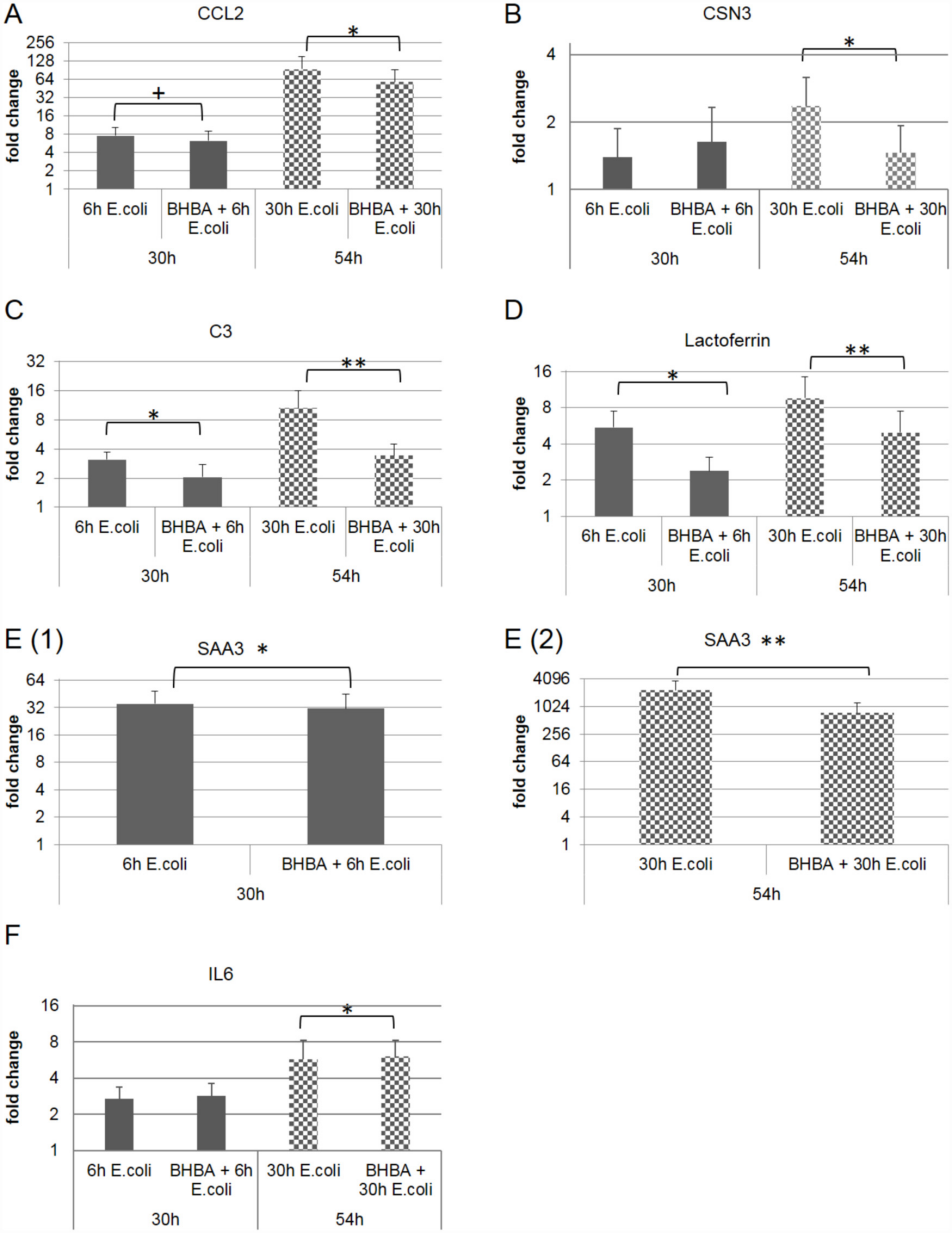
Attenuation of the gene expression through the co-stimulation of pbMEC with *E*. *coli* and 3 mM BHBA. Fold change of **(A)** the chemokine CCL2, **(B)** the milk protein CSN3, **(C)** the complement component C3, **(D)** the antimicrobial peptide LF, **(E 1–2)** the acute phase protein SAA3, and **(F)** the inflammatory cytokine IL6. Significant changes in the gene expression between the *E*. *coli* treated pbMEC and the pbMEC co-stimulated with *E*. *coli* and 3 mM BHBA are indicated by stars: *p ≤ 0.05, **p ≤ 0.01, ***p ≤ 0.001.

The direct comparison of the *E*. *coli* treatment and the co-stimulation with 3 mM BHBA and *E*. *coli* indicated a significant down-regulation of the gene expression within the pbMEC that received challenge medium supplemented with *E*. *coli* and 3 mM BHBA. The p-values were evaluated using a normal t-test that compared the dCq values of the *E*. *coli* treated pbMEC directly to the dCq values of the co-stimulated pbMEC (Fig 3). Within the genes coding for the chemokines, the gene coding for CCL2 showed a distinct down-regulation 6 h post-infection. There was also a significant attenuation of gene expression 30 h after treatment start in the co-stimulatory group (Fig 3A). The gene expression of IL6 was the only one that was significantly induced due to the co-stimulation 30 h post-infection when directly compared to pbMEC, which were only treated with *E*. *coli* for 30 h (Fig 3F). The genes coding for the antimicrobial peptide LF, the acute phase protein SAA3, and the complement factor C3 showed the same trend when the p-values were calculated (Fig 3C, 3D, 3E(1) and 3E(2)). All three genes showed a significant and a highly significant down-regulation of the gene expression when pbMEC were treated with both *E*. *coli* and 3 mM BHBA (Fig 3C, 3D, 3E(1) and 3E(2)). Even if no changes in the gene expression of the gene coding for the milk protein CSN3 could be detected through the *E*. *coli* stimuli itself, the gene expression of CSN3 in the co-stimulatory experiment was significantly down-regulated 30 h after treatment start compared to the treatment with only *E*. *coli* (Fig 3B).

### Correlation of RT-qPCR data with protein data obtained from ELISAs of the cell culture supernatants

**Fig 4:**
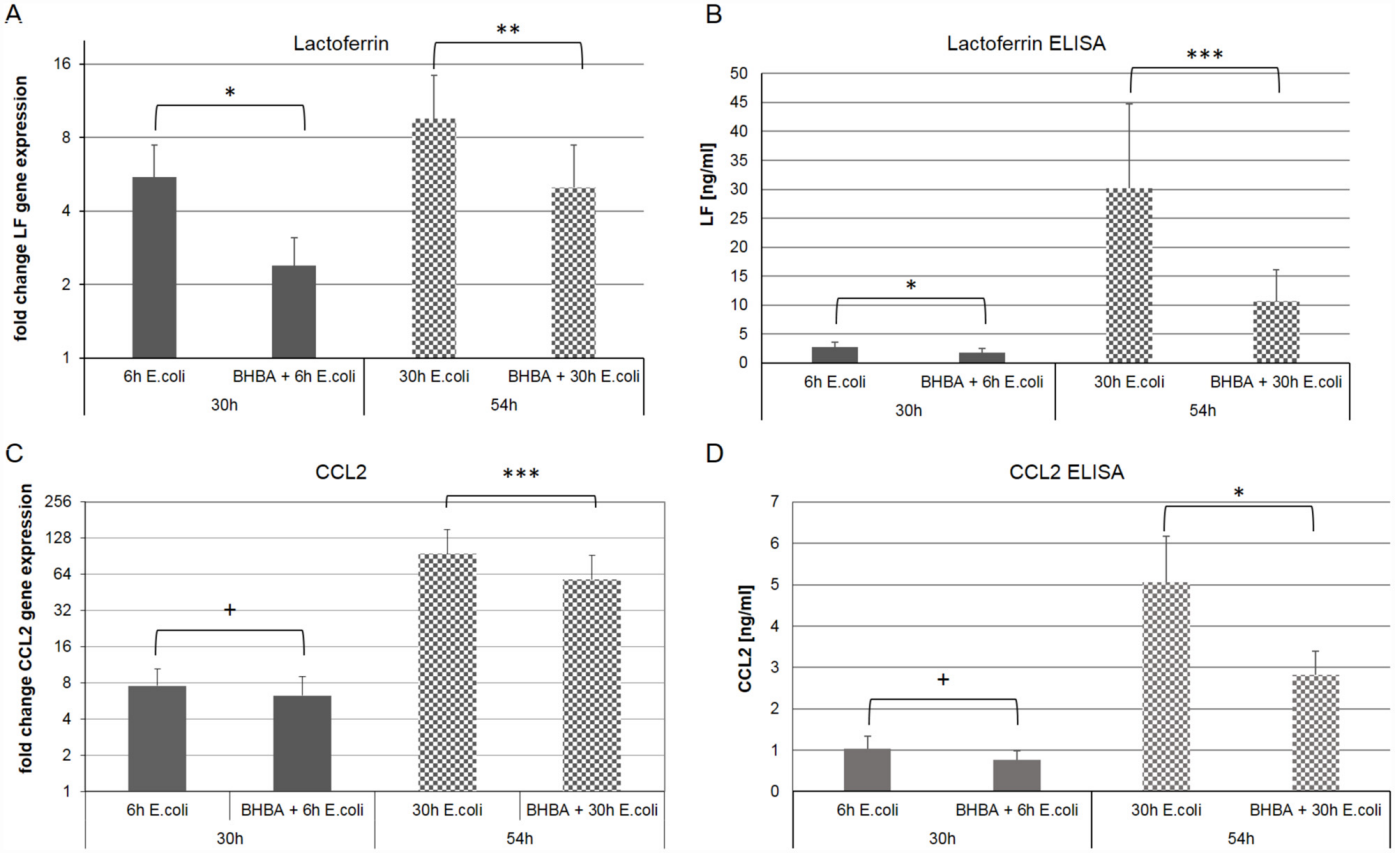
Comparison of the RT-qPCR data of LF and CCL2 with the LF and CCL2 content in pbMEC cell culture supernatants. The fold changes of the LF gene expression (A) indicated a significant down-regulation of LF gene expression when pbMEC where co-stimulated with *E*. *coli* and 3 mM BHBA. The same effect could be detected within the competitive LF ELISA (B) of pbMEC cell culture supernatants. The amount of secreted LF decreased significantly in case of the co-stimulation. The distinct and significant down-regulation in CCL2 gene expression (C) could also be confirmed by the results of the bovine CCL2 VetSet^™^ ELISA Kit (D). The CCL2 gene expression and the amount of secreted protein decreased distinctly as well as significantly within the co-stimulatory group. Significant changes: *p ≤ 0.05, **p ≤ 0.01, ***p ≤ 0.001, distinct changes: + 0.1 ≤ p ≤ 0.05.

For LF and CCL2, the RT-qPCR results could also be validated with an ELISA measurement. The LF content in cell culture supernatants could be detected using the competitive LF-ELISA, which is routinely used at our institute (S3 Table). The LF content in the cell culture supernatants increased upon treatment of the pbMEC with the mastitis pathogen *E*. *coli*. However, the amount of secreted LF decreased significantly 6 h and 30 h after treatment start within the cell culture supernatant of the co-stimulated pbMEC (Fig 4B). The same trend could be seen when the RT-qPCR data of CCL2 (Fig 4C, S4 Table) was compared to the protein data obtained by the bovine CCL2 VetSet^™^ ELISA Development Kit. The amount of secreted CCL2 increased upon stimulation with the gram-negative pathogen *E*. *coli*, but decreased distinctly and significantly in the cell culture supernatant of the co-stimulated pbMEC (Fig 4D), thus confirming the results obtained by RT-qPCR.

## Discussion

Dairy cows are often challenged by mastitis-inducing gram-negative pathogens, like *E*. *coli*. Despite several physiological defense barriers of the bovine mammary gland, those pathogens are likely to enter the blood circulation via pbMEC, which are part of the blood-mammary gland barrier [20,21]. Therefore, it has already been shown in previous studies that pbMEC are mandatory for the induction of the innate immune response of the bovine mammary gland [22]. It has already been proven that pbMEC bear germline-encoded TLR on their cell surface and are hence able to directly respond to invading pathogens, due to the recognition of so-called pathogen-associated molecular patterns (PAMP) [11,22]. It is known that innate leukocytes are one of the most essential cellular components of innate immunity but bovine innate leukocytes—and especially phagocytic neutrophils and macrophages—are not directly attracted to pathogens or bacterial products [23,24]. Therefore, the induction of the TLR signaling pathway that leads to the activation of the pleiotropic transcription factor NFĸB and hence to the induction of the gene expression of genes coding for intracellular signaling molecules and chemotactic molecules, like pro-inflammatory cytokines, chemokines, components of the complement system and acute phase proteins, is crucial for the recruitment of leukocytes to the site of inflammation [25,26]. As several previous studies [9,11,22,26] have already shown that bacterial challenge of pbMEC *in vitro* resulted in differential gene expression of the above-mentioned innate immune gene classes, we also focused on those gene expression profiles. We wanted to elucidate whether the treatment with *E*. *coli* and/or BHBA could interfere with the gene expression of components of the MYD88 dependent TLR4 signaling cascade that is responsible for the proper activation of the essential p65 NFĸB transcription factor subunit RELA. Furthermore, as it is known that the danger associated molecular pattern molecule S100A9 and chemokines, like CCL2, CCL20 and especially CXCL8, are important mediators of the inflammatory response, we also investigated changes in the gene expression profile of components of those innate immune gene families [9,22,25,27,28]. Especially CXCL8 is known to be essential for the recruitment of leukocytes into the bovine mammary gland [25,29]. Furthermore, CCL2 as well as CXCL8 are discussed to induce locally restricted polarized diapedesis of neutrophils across epithelia and endothelia [9,30]. Furthermore, S100A9 is also discussed to contribute to the recruitment of leucocytes [28]. Despite of the chemokines, we were also interested in changes in the gene expression of genes coding for selected pro-inflammatory cytokines, like IL6 and TNFα. For both, IL6 and TNFα, it has been postulated that they act as acute phase cytokines and therefore are of great importance to the recruitment and activation of neutrophils [31,32]. We further focused on the gene expression of innate immune genes coding for components of the innate humoral defense line. Among them were genes coding for the complement component C3, the acute phase protein SAA3, several β-defensins (HP, TAP, LAP) and antimicrobial peptides (LYZ1, LF). Those genes are normally constitutively expressed, even if no bacterial stimuli is present but can be significantly induced upon pathogen recognition [22,25]. Those humoral defense molecules are more likely to directly act on the pathogen, while also contributing to the chemotactic gradient like the other classes of innate immune genes presented in this paragraph. It has been shown that LF, LYZ1 TAP, LAP and HP exhibit bacteriostatic and bactericidal properties [11,25]. Furthermore, the proper functionality of the pbMEC used in this study was monitored by the evaluation of the gene expression profile of genes involved in milk protein synthesis (STAT5A, β-casein, ĸ-casein).

### The innate immune response of pbMEC is strongly induced by *E*. *coli*

Innate pathogen recognition is mainly mediated by the toll-like receptor pathway. In the present study, TLR4 and the signal transducer MYD88 were analyzed. Both were expressed in our pbMEC proving their ability to perceive invading pathogens. However, the expression of TLR4 and MYD88 was largely unaffected by the *E*. *coli* challenge, although there was a pronounced inflammatory response. Similarly, [10] found no effect of LPS on TLR4 expression in pbMEC and concluded that pbMEC contain a fully functional and constitutively active TLR signaling pathway, which does not need to be up-regulated upon pathogen invasion. It seems that the continuously expressed toll-like receptors are sufficient to induce an adequate inflammatory response by the activation of signaling cascades that lead to the production of cytokines and chemokines [10]. RELA, a subunit of NF-κB, was also not differentially expressed in *E*. *coli* treated pbMEC. This can be explained by its role as a signal transducer, which needs to be constitutively expressed in order to response immediately upon pathogen invasion.

The genes coding for chemokines like CCL2, CCL20 and CXCL8 were significantly up-regulated in pbMEC stimulated with *E*. *coli*. The same effect could be found for the pro-inflammatory cytokines TNFα and IL6. This confirmed the contribution of pbMEC to the innate immune system, as they exert immune modulatory functions. Following pathogen invasion, pbMEC secrete signaling molecules like cytokines and chemokines to induce an inflammatory response and to recruit immune effector cells like monocytes and lymphocytes to the site of infection [33]. Our results are consistent with other studies which already mentioned the major role of pbMEC in triggering the innate immune response of the bovine mammary gland [9,13,22]. It has been shown [34] that cell culture supernatants of *E*. *coli* treated pbMEC enhanced the chemotactic activity of leukocytes, which is most likely a direct effect of the induced chemokine production.

Exposure to *E*. *coli* activated the gene expression of genes coding for the antimicrobial peptides LF, LYZ1 and TAP. The acute phase protein SAA3 and the complement component C3 were also induced by *E*. *coli*. This is in accordance with published data [12], where *E*. *coli* also caused a strong induction of the gene expression of those defense molecules. The antimicrobial peptide LF is known to inhibit the iron-dependent bacterial growth by binding free iron in milk [25] and it is also able to directly kill bacteria by disrupting their cell membrane [35], whereas LYZ1 destroys the outer membrane of gram-negative bacteria, like *E*. *coli*, by cleaving peptidoglycans [36]. The β-defensin TAP and the acute phase protein SAA3 exert antimicrobial functions as well. SAA3, for instance, facilitates phagocytosis by opsonizing gram-negative bacteria [37]. Activation of the complement cascade is another defense mechanism, as it results in the formation of the membrane attack complex that disrupts the bacterial cell membrane. Cleavage products of complement component C3 exert antimicrobial activity and are able to opsonize microbes, thereby facilitating phagocytosis [38]. However, the most up-regulated gene upon *E*. *coli* treatment was SAA3. The strong induction of the acute phase protein upon pathogen invasion has also been shown in a study of [9,22,27]. SAA3 has already been proposed as a potential biomarker for mastitis [27]. Furthermore, the gene coding for the danger associated molecular pattern molecule S100A9 tended to be up-regulated by the *E*. *coli* treatment, but fold changes were not significant. S100A9 belongs to the S100 calgranulins, a group of anti-infective and anti-inflammatory proteins. Their functions include recruitment of leukocytes, antimicrobial activity and oxidant scavenging [28].

### The presence of BHBA attenuates the innate immune response of pbMEC to *E*. *coli*

The present study is one of the first studies investigating the exclusive effect of the ketone body BHBA on the innate immune response of pbMEC to *E*. *coli in vitro*. Prior to the immune challenge with the mastitis pathogen *E*. *coli*, pbMEC were accustomed to the metabolic state of ketosis by exposure to 3 mM BHBA for 24 h. During the immune challenge, the exposure to BHBA was continued. This approach was chosen because we wanted to analyze the influence of an already existing ketosis on the innate immune response of pbMEC. It has been shown in epidemiological studies that there is indeed an association between ketosis and an increased risk of mastitis [39–41]. It is not yet clear by which mechanism ketosis interferes with the immune defense of the bovine mammary gland. However, elevated BHBA levels are considered to have inhibiting effects on immune cells [1]. Our *in vitro* approach aimed to investigate whether this also applies to the innate immune response of bovine mammary epithelial cells. In the present study, 4 of the 15 analyzed innate immune genes showed a significant down-regulation in pbMEC co-stimulated with *E*. *coli* and 3 mM BHBA. Those were genes coding for the chemokine CCL2, the acute phase protein SAA3, the antimicrobial peptide LF and the complement component C3. ELISA measurements for LF and CCL2 confirmed the finding of the RT-qPCR (Fig 4A, S3 and S4 Tables). Therefore, protein biosynthesis followed the same trend as gene expression when the ketone body BHBA was present in the cell culture supernatant. The only gene that showed a significant induction in gene expression in the co-stimulatory experiment was the pro-inflammatory cytokine IL6. Interestingly, the innate immune genes influenced by BHBA belonged to different gene families, and genes from almost every analyzed gene family were affected by the BHBA treatment in the co-stimulatory approach. This leads to the conclusion that the immunosuppressive effect of BHBA results from an overall suppression of the immune response and is not restricted to a single group of immune response genes. In general, the immunosuppressive effect of BHBA was more pronounced after 54 h, indicating that especially long-term ketosis had a negative effect on the gene expression of innate immune genes. These findings are in agreement with a study on the influence of BHBA on the chemotactic functions of leukocytes [42]. The authors observed an impairment of the chemotactic capacity of leukocytes from naturally-occurring ketotic cows.

We could show that stimulation of pbMEC with the ketone body BHBA not only attenuates the gene expression of the genes coding for CCL2 and LF, but also their protein biosynthesis and hence protein secretion into the cell culture medium. The reduced concentrations of the chemotactic protein CCL2 and the humoral response protein LF, are very likely to lead to a reduced lymphocyte chemotaxis towards the bovine mammary gland, leading to greater mastitis susceptibility in cows.

A previously published study investigated the innate defense capability of pbMEC, which were isolated after an induced negative energy balance *in vivo*, and subsequently challenged with *E*. *coli in vitro* [13]. In this study, the combination of *E*. *coli* treatment and the dietary energy deficit induced generally higher expression levels compared to the control-fed group. This is contrary to our findings, as we observed a down-regulation of the gene expression by co-stimulation of pbMEC with *E*. *coli* and 3 mM BHBA. However, the restriction feeding in the above-mentioned experiment did not induce ketosis as proven by the small change in plasma BHBA concentrations of restricted cows compared to the control cows [13,25,43]. Additionally, pbMEC were cultivated in a common proliferation medium without BHBA, so that they were likely to regenerate from the previously experienced NEB during cultivation. In contrast, our *in vitro* approach guaranteed a constant concentration of 3 mM BHBA during the whole experiment, as the BHBA containing challenge medium was renewed every day.

## Conclusion

The present *in vitro* study aimed to investigate the influence of ketosis on the innate immune response of the bovine mammary gland to mastitis. Therefore, pbMEC isolated from milk were challenged with the ketone body BHBA and the mastitis pathogen *E*. *coli*. Subsequently, the changes in gene expression of innate immune genes were determined via RT-qPCR. An explicit advantage of the present *in vitro* approach was the fact that it enabled the investigation of the specific effect of BHBA on the innate immune response. Furthermore, experimental conditions like BHBA concentration could be exactly defined. Exposure of pbMEC to the mastitis pathogen *E*. *coli* strongly induced the gene expression of several genes related to the innate immune response like cytokines, chemokines, antimicrobial peptides, the complement factors and the acute phase proteins. This proves the importance of pbMEC in the induction and promotion of the innate immune response of the bovine mammary gland. Besides their ability to directly combat invading pathogens by secreting various defense molecules, pbMEC are also responsible for the recruitment of immune cells to the site of infection. The results of our study indicated that increased BHBA concentrations may be at least partially responsible for the higher mastitis susceptibility of dairy cows in early lactation. The gene expression of CCL2, SAA3, LF and C3 was significantly down-regulated in pbMEC co-stimulated with 3 mM BHBA and *E*. *coli* compared to the gene expression of pbMEC stimulated only with *E*. *coli*. Hence, the immunosuppressive effect of BHBA might be attributed to an overall suppression of immune response genes. Long-term elevated BHBA concentrations, in particular, seem to have a negative effect on the innate immune response.

## Supporting Information

S1 Table Fold change data for Table 2. (XLSX)

S2 Table Differences in the gene expression of pbMEC treated with *E*. *coli* or with *E*. *coli* and 3 mM BHBA. (DOCX)

S3 Table LF ELISA data for generation of Fig 4. **(A)** Data for 6 h *E*. *coli* treatment and 30 h of co-stimulation. **(B)** Data for 30 h *E*. *coli* treatment and 54 h of co-stimulation. **(C)** Standard curve.
(XLSX)

S4 Table CCL2 ELISA data for generation of Fig 4. **(A)** Data for 6 h *E*. *coli* treatment and 30 h of co-stimulation. **(B)** Data for 30 h *E*. *coli* treatment and 54 h of co-stimulation. **(C)** Standard curve.
(XLSX)
