# Supplementary material for: Effect of the Ketone Body Beta-Hydroxybutyrate on the Innate Defense Capability of Primary Bovine Mammary Epithelial Cells
Source: PLoS One. 2016 Jun 16;11(6):e0157774. doi: 10.1371/journal.pone.0157774 (PMC4910980; doi:10.1371/journal.pone.0157774)
Supplement: S2 Table — (DOCX) [file pone.0157774.s002.docx]

**S2 Table:** Differences in the gene expression of pbMEC treated with E. coli or with E. coli and 3 mM BHBA

|  |  | Time-point | | | | | | |  |
| --- | --- | --- | --- | --- | --- | --- | --- | --- | --- |
|  |  | 30 h | | | 54 h | | | |  |
|  |  | treatment | | | treatment | | | |  |
| genes |  | *E.coli^1^* | BHBA+*E.coli^2^* |  | *E.coli^3^* | BHBA+*E.coli^4^* |  | |  |
| ***Chemokines*** | | | | | | | | |  |
| CCL2 | Fold | 7.56 | 6.29 | + | 94.72 | 58.12 | | * | |
|  | SEM | 2.00 | 2.12 |  | 46.64 | 27.19 | |  | |
| ***Inflammatory cytokines*** | | | | | | | | |  |
| IL6 | Fold | 2.71 | 2.86 |  | 5.72 | 6.07 | * | |  |
|  | SEM | 0.49 | 0.61 |  | 1.99 | 1.86 |  | |  |
| ***Antimicrobial peptides*** | | | | | | | | |  |
| LF | Fold | 5.49 | 2.39 | * | 9.59 | 4.99 | ** | |  |
|  | SEM | 1.85 | 0.68 |  | 4.32 | 2.04 |  | |  |
| ***Acute phase proteins*** | | | | | | | | |  |
| SAA3 | Fold | 35.19 | 31.38 | * | 2302.14 | 738.94 | ** | |  |
|  | SEM | 11.15 | 13.04 |  | 1183.19 | 361.50 |  | |  |
| ***Complement system*** | | | | | | | | |  |
| C3 | Fold | 3.12 | 2.04 | * | 10.54 | 3.43 | ** | |  |
|  | SEM | 0.60 | 0.62 |  | 4.72 | 0.84 |  | |  |
| ***Lactogenesis*** | | | | | | | | |  |
| CSN3 | Fold | 1.40 | 1.64 |  | 2.36 | 1.46 | * | |  |
|  | SEM | 0.35 | 0.57 |  | 0.69 | 0.37 |  | |  |

^1^: 6 h *E. coli* treatment

^2^: 30 h BHBA and 6 h *E. coli* treatment

^3^: 30 h *E. coli* treatment

^4^: 54 h BHBA and 30 h *E. coli* treatment

+: Trend 0.1 ≤ p ≤ 0.05

*p ≤ 0.05

**p ≤ 0.01
